# Pretreatment neutrophil to lymphocyte ratio in determining the prognosis of head and neck cancer: a meta-analysis

**DOI:** 10.1186/s12885-018-4230-z

**Published:** 2018-04-04

**Authors:** Yalian Yu, Hongbo Wang, Aihui Yan, Hailong Wang, Xinyao Li, Jiangtao Liu, Wei Li

**Affiliations:** 1 Department of Otorhinolaryngology, the First Affiliated Hospital of China Medical University, Shenyang, Liaoning Province People’s Republic of China; 2 Department of Radiology, Shengjing Hospital of China Medical University, Shenyang, Liaoning Province People’s Republic of China; 3 Department of Clinical Epidemiology and Center of Evidence Based Medicine, the First Affiliated Hospital of China Medical University, Shenyang, Liaoning Province People’s Republic of China; 4 Department of Cardiovascular Surgery & Electro-chemotherapy, China-Japan Friendship Hospital, Beijing, People’s Republic of China

**Keywords:** Head and neck cancer, Neutrophil-to-lymphocyte ratio, Prognosis, Meta-analysis

## Abstract

**Background:**

Recent studies have reported a relationship between prognosis and neutrophil-to-lymphocyte ratio (NLR) in patients with head and neck cancer (HNC). As the results are still controversial, we conducted a meta-analysis of pretreatment NLR in peripheral blood and prognosis in HNC patients.

**Methods:**

We retrieved articles from PubMed, Medline, Cochrane Library, Embase and Web of Science. A comparative analysis was conducted for the effect of pretreatment NLR in peripheral blood on overall survival (OS), progression-free survival, disease-free survival (DFS), disease-specific survival, metastasis-free survival, and recurrence-free survival of HNC patients. The analysis applied the criteria for systematic reviews described in the Cochrane Handbook and was conducted using hazard ratios (HRs) to estimate effect size, and calculated by Stata/SE version 13.0.

**Results:**

The meta-analysis included eligible cohort studies (5475 cases). The OS data indicated increased mortality risk in HNC patients with a high NLR (HR = 1.84, 95% confidence interval (CI): 1.53–2.23; *P* < 0.001; heterogeneity, I^2^ = 37.2%, *P* = 0.074). Analysis of subgroups stratified by NLR cutoff values revealed increased mortality risk and significantly shorter DFS in patients with high NLR compared to those with low NLR (HR = 2.18, 95% CI: 1.46–3.24; *P* < 0.001). Patients with high NLR had a higher probability of tumor recurrence after treatment than those with low NLR (HR = 1.63, 95% CI: 1.09–2.45; *P* = 0.017; heterogeneity, *I*^2^ = 68.7%; *P* = 0.022). The probability of distant metastasis following treatment was greater in patients with high compared with low NLR (HR = 1.92, 95% CI: 1.36–2.72; *P* < 0.001; heterogeneity, *I*^2^ = 0.0%; *P* = 0.614). Funnel plots of the meta-analysis results were stable, as shown by sensitivity analysis. No publication bias was detected by the Egger test (*P* = 0.135).

**Conclusions:**

HNC patients with elevated pretreatment NLR in peripheral blood have poor prognosis and are prone to local invasion and distant metastasis. NLR values are easily obtained from routinely collected blood samples and could assist clinicians to determine prognosis of HNC patients.

## Background

Head and neck cancer (HNC) is currently the fifth most common malignancy worldwide, with > 600,000 new cases and > 300,000 deaths annually [1, 2]. Despite effective surgical interventions and adjuvant therapy, the 5-year HNC survival rate of nearly 50% is still lower than that of most other cancers [3]. HNC originates in the mucosal epithelium of the oropharynx, nasopharynx, nasal and paranasal sinuses, larynx and hypopharynx. Many patients are diagnosed with HNC at an advanced stage. Data from the United States show that more than two-thirds of HNC patients present with lymph node invasion or distant metastasis at the time of diagnosis. More than half the patients need more surgery or radiotherapy because of recurrence within 2 years of initial surgery [4]. Therefore, simple, effective and economically feasible laboratory indices that can predict increased risk of recurrence, metastasis or death in HNC patients are essential for early diagnosis and improved survival in clinical practice.

Awareness of the presence of inflammation in the tumor microenvironment has spurred research on the relationship between inflammation and malignancy [5–10]. The progression of cancer requires interactions between tumor cells and their microenvironment, including inflammatory, immune and metabolic responses to stimuli from the surrounding tissue. The systemic inflammatory response plays a key role in tumor cell invasion by promoting microvascular regeneration, tumor metastasis, and tumor cell proliferation [8, 9, 11]. Moreover, the systemic inflammatory response facilitates the differentiation of tumor cells and suppresses activity of host immune cells [6, 12–14]. Neutrophil-to-lymphocyte ratio (NLR) is an accurate and reliable index of systemic inflammation. NLR is closely associated with prognosis of solid tumors, such as colorectal, non-small cell lung, stomach and prostate cancer [15–19]. However, the association of NLR and prognosis of HNC remains controversial. For that reason, we conducted this meta-analysis of the prognostic value of NLR in HNC.

## Methods

### Literature search strategy

A systematic research was performed according to the Preferred Reporting Items for Systematic Reviews and Meta-Analysis (PRISMA) guidelines [20]. The research of PubMed, Embase, Cochrane Library and Web of Science identified relevant studies published in English or Chinese up to June 2016. The search strings included “head and neck cancer”, “head and neck carcinoma”, “head and neck neoplasms”, “neutrophil-to-lymphocyte ratio”, “neutrophils”, “lymphocytes”, and “NLR”. Manual searches of reference lists in articles retrieved online were conducted to identify additional relevant studies.

### Literature inclusion and exclusion criteria

Articles were included following independent searches by two of the authors (YY and XL). Disagreements were resolved by discussion or intervention by a third researcher (HW). To be included, a study had to report findings on the association between prognosis in head and neck tumors and NLR in peripheral blood before therapeutic intervention. The interventions included surgical resection, radiotherapy, chemotherapy, or combined therapy. Prognosis-related survival data included hazard ratio (HR) with 95% confidence intervals (CIs), or curves of overall survival (OS), progression-free survival (PFS), disease-free survival (DFS), disease-specific survival (DSS), metastasis-free survival (MFS), or recurrence-free survival (RFS). Studies were excluded using the criteria of the Cochrane Nonrandomized Studies Methods Group [21]. Duplicate reports and duplicate cases (with multiple reports of the same study, the most recent publication was selected), and case reports were excluded. We also excluded articles without available full text; articles with incomplete survival data that could not be obtained following communication with the authors; literature reviews; and conference abstracts that lacked sufficient data for meta-analysis quality assessment.

### Data extraction

We extracted data indicating country or region, author, title, year of publication, journal name, postal or e-mail address, type of research, sample size, age, gender, intervention measure, tumor type, AJCC or UICC cancer stage, lesion site, duration of or lost to follow-up, HR and 95% CI, and NLR cutoff value used to define OS, PFS and DFS. For studies that lacked complete data, the results of multivariate analysis were preferable to those of univariate analysis, but in the absence of multivariate analysis, univariate analysis was accepted. If HRs were not presented, they were calculated from the survival curve data as described by Tierney et al. [22].

### Assessment of included studies

There are no criteria for evaluation of treatment described in prognostic cohort studies included in systematic reviews. Consequently, each study was assessed by two researchers (YY and XL) following the Newcastle–Ottawa Scale (NOS) for assessing the quality of cohort studies [23]. The maximum NOS score is 9 points, and studies with scores > 5 points were classified as high quality. Disagreements were resolved by discussion with a third researcher (HW).

### Statistical analysis

Data analysis and processing were carried out using Stata/SE version 13.0 (StataCorp LP, College Station, TX, USA) provided by the Cochrane Collaboration. Disagreements were resolved through discussion. OS, PFS and DFS were evaluated using HR and 95% CI to describe the size of the treatment effect. χ^2^ tests were conducted at α = 0.05, with *P* < 0.1 as significant. The measure of heterogeneity was *I*^2^, and < 25% indicated low heterogeneity, 25–50% indicated moderate heterogeneity, and > 50% indicated high heterogeneity. A fixed effects model was used for studies without heterogeneity, and a random effects model was used for studies with heterogeneity. Meanwhile, subgroup analysis and meta-regression methods were used for heterogeneity analysis. Publication bias was assessed by the Egger test using Stata/SE version 13.0, and the results are shown in funnel plots. Sensitivity analysis was conducted by the meta-trim method.

## Results

### Included studies and quality assessment

**Fig. 1:**
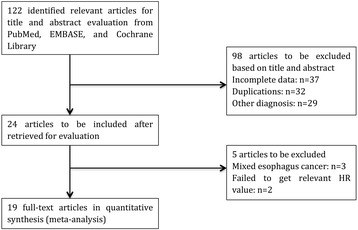
Literature screening flowchart

A total of 122 relevant studies were retrieved; 98 of which were excluded at the initial assessment of titles and abstracts, and the full-text of the remaining 24 was further screened. Nineteen eligible nonrandomized studies [24–42], all of which were cohort studies and included a total of 5475 patients, were included in the analysis. A flowchart of the inclusion and exclusion criteria of each study is shown in Fig. 1. Two researchers agreed on the 19 studies that were finally selected. All studies included patients with pretreatment NLR and survival data, and the study data and quality assessment results of each study are summarized in Table 1. The Cox regression hazard model used to adjust for potential confounding bias included the majority but not all of the included studies. If multivariate analysis of survival data was unavailable, univariate analysis was adopted for assessment of the survival data. The HNC tumor sites included the mouth, nasal and paranasal sinuses, nasopharynx, larynx and hypopharynx.

**Table 1:** Characteristics and quality assessment results for each included publications

Study	Country	Ethnicity	Tumors	Patients(female/male)	Age(range)	Result	Follow-up (month)	Uni\Multi	Cutoff value	NOS Score
Sun et al, 2016 [24]	China	Asian	NC	251 (71\180)	46 (15-76)	OS PFS	50 (5-84)	Multi	2.6	8
Wong et al, 2015 [25]	UK	Caucasian	LSCC	140 (19\121)	66 (36-92)	OS DFS	41 (2-103)	Multi	3.1	8
Fu et al, 2016 [26]	China	Asian	LSCC	420 (7\413)	60 (33-84)	OS CSS	ungiven	Multi	2.59	7
An et al, 2011 [27]	China	Asian	NC	363 (89\274)	47 (12-76)	DSS MFS	62 (2-92)	Multi	3.73	7
Li et al, 2015 [28]	China	Asian	NC	363 (89\274)	47 (12-76)	DSS	14.7 (3.22-92.9)	Multi	2.81	8
He et al, 2012 [29]	China	Asian	NC	1410 (383\1027)	46.1 (13-79)	OS PFS	41 (2-60)	Multi	2.74	7
Fang et al, 2013 [30]	China	Asian	OCSCC	226 (19\207)	52.47 (27.0-84.0)	OS DFS	ungiven	Uni	2.44	6
Nakahira et al, 2016 [31]	Japan	Asian	NS	100 (14\86)	65.2 (37-85)	CSS	37.85 (4-92)	Multi	3	8
Perisanidis et al, 2013 [32]	Austria	Caucasian	OCSCC	97 (30\67)	ungiven	DSS	> 5 years or until death	Multi	1.9	7
Charles et al, 2016 [33]	Australia	Caucasian	HNSCC	145 (30\115)	63 (23-86)	OS RFS	29 (1.5-84)	Multi	5	8
Tu et al, 2015 [34]	China	Asian	LSCC	141 (4\137)	59 (36-87)	OS DFS	51 (5-102)	Multi	2.17	7
Moon et al, 2016 [35]	Korea	Asian	HNSCC	153 (24\129)	57 (16-78)	OS PFS CSS	39.5 (4.7-62.6)	Multi	3.3	8
Rachidi et al, 2016 [36]	America	Caucasian	HNSCC	543 (123\420)	58.8	OS	64.4 (2-156)	Multi	4.39	8
Song et al, 2015 [37]	China	Asian	HSCC	146 (10\136)	57.5 (34-89)	OS	33.2 (2-128)	Uni	2.3	7
Salim et al, 2015 [38]	Turkey	Caucasian	HNSCC	79 (8\71)	59 (28-85)	OS	ungiven	Uni	2.93	6
Haddad et al, 2015 [39]	Australia	Caucasian	HNC	46 (8\38)	59 (43-81)	OS MFS RFS	34 (13-47)	Uni	5	5
Rassouli et al, 2015 [40]	Canada	Caucasian	HNSCC	273 (75\198)	64 + _12	DFS	45 (42-48)	Uni	4.27	6
Selzer et al, 2015 [41]	Austria	Caucasian	HNC	318 (121\247)	ungiven	OS	ungiven	Uni	1.58	7
Kim et al, 2016 [42]	Korea	Asian	HNC	104 (9\95)	58 (20-82)	OS PFS	39 (4.8-82.5)	Multi	3	8

*NC* nasopharyngeal carcinoma, *LSCC* laryngeal squamous cell carcinoma, *OCSCC* Oral Cavity Squamous Cell Carcinoma, *HNSCC* Head and neck squamous cell carcinoma, *HSCC* hypopharyngeal squamous cell carcinoma, *HNC* head and neck cancer, *Uni* univariate analysis, *MFS* metastasis-free survival, *Multi* multivariate analysis, *NOS score* Newcastle-Ottawa Scale score, > 5 meant relative good quality

### OS of HNC patients and subgroup analysis by NLR cutoff value

**Fig. 2:**
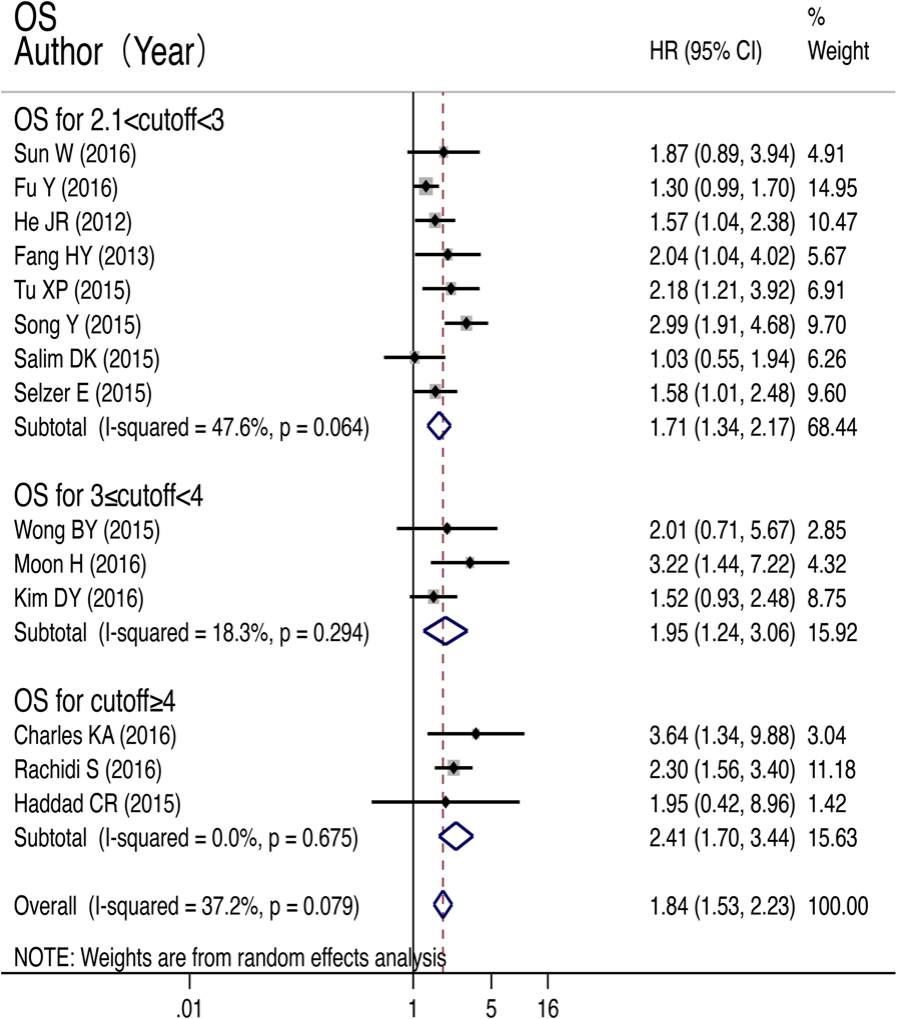
Forest plots of studies evaluating HRs of the NLR on OS and subgroup based on cutoff value

Fourteen studies were included in the meta-analysis of OS. The mortality risk of patients with high NLR was 1.84 times that of patients with low NLR. The difference was significant (HR = 1.84, 95% CI: 1.53–2.23; *P* < 0.001; heterogeneity, *I*^2^ = 37.2%, *P* = 0.074; Fig. 2a). Subgroup analysis by NLR cutoff value revealed a higher mortality risk in patients with high NLR compared to those with low NLR. The difference reached significance (2.1 < cutoff < 3, HR = 1.71, 95% CI: 1.34–2.17, *P* < 0.001; heterogeneity, *I*^2^ = 47.6%, *P* = 0.064; 3 ≤ cutoff < 4, HR = 1.94, 95% CI: 1.235–3.064, *P* = 0.005; heterogeneity, *I*^2^ = 18.3%, *P* = 0.294; cutoff ≥4, HR = 2.414, 95% CI: 1.696–3.436, *P* < 0.001, heterogeneity: *I*^2^ = 0.0%, *P* = 0.675; Fig. 2b). In the subgroup-analysis of ethnicity, either Asian [24, 26, 29, 30, 34, 35, 37, 42] or Caucasian [25, 33, 36, 38, 39, 41] patients with an evaluated indicated NLR a poor predictor of overall survival. All the results above are shown in Table 2.

**Table 2:** Summary of meta-analysis results

Outcomes	Variable	N	References	Fixed-effect model		Random-reffect model		Heterogeneity	
				HR(95%CI)	p	HR(95%CI)	p	I2	p
OS	Ethnicity								
	Asian	8	[24, 26, 29, 30, 34, 35, 37, 42]	1.72 (1.46,2.03)	< 0.001	1.87 (1.46,2.40)	< 0.001	49%	0.056
	Caucasian	6	[25, 33, 36, 38, 39, 41]	1.85 (1.44,2.37)	< 0.001	1.83 (1.34,2.51)	< 0.001	26%	0.239
	cutoff value								
	2.1 < cutoff< 3	8	[24, 26, 29, 30, 34, 37, 38, 41]	1.63 (1.39,1.91)	< 0.001	1.71 (1.43,2.17)	< 0.001	47.60%	0.064
	3 < =cutoff< 4	3	[25, 35, 42]	1.88 (1.28,2.77)	0.001	1.95 (1.24,3.06)	0.04	18.30%	0.249
	cutoff> = 4	3	[33, 36, 39]	2.41 (1.70,3.44)	< 0.001	2.41 (1.70,3.44)	< 0.001	0	0.675
DFS		4	[25, 30, 34, 40]	1.99 (1.46,2.71)	< 0.001	1.99 (1.46,2.71)	< 0.001	0	0.457
MFS		3	[27, 28, 39]	1.92 (1.36,2.72)	< 0.001	1.92 (1.36,2.72)	< 0.001	0	0.614
PFS		5	[24, 29, 35, 38, 42]	1.60 (1.37,1.87)	< 0.001	2.17 (1.20,3.92)	0.01	91.30%	0

### PFS and DFS for HNC patients

**Fig. 3:**
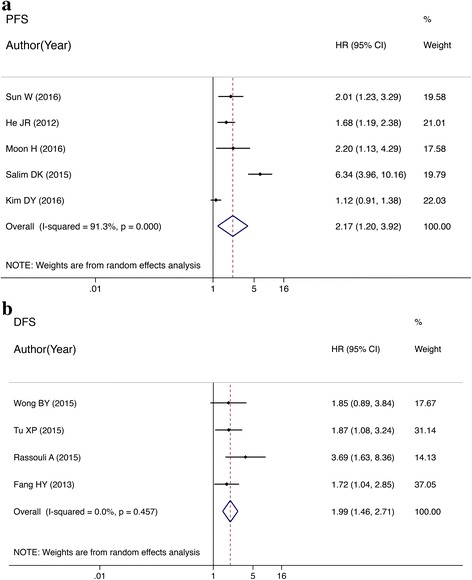
**a** Forest plots of studies evaluating HRs of the NLR on PFS for head and neck cancer. **b** Forest plots of studies evaluating HRs of the NLR on DFS for head and neck cancer

The meta-analysis of PFS showed that malignancy was more likely to progress in patients with high NLR than in those with low NLR. The difference was significant (HR = 2.17, 95% CI: 1.20–3.92; *P* < 0.001; Fig. 3a). Patients with high NLR had shorter DFS than those with low NLR. The difference reached significance (HR = 2.18, 95% CI: 1.46–3.24; *P* < 0.001; Fig. 3b).

### RFS and MFS for HNC patients

**Fig. 4:**
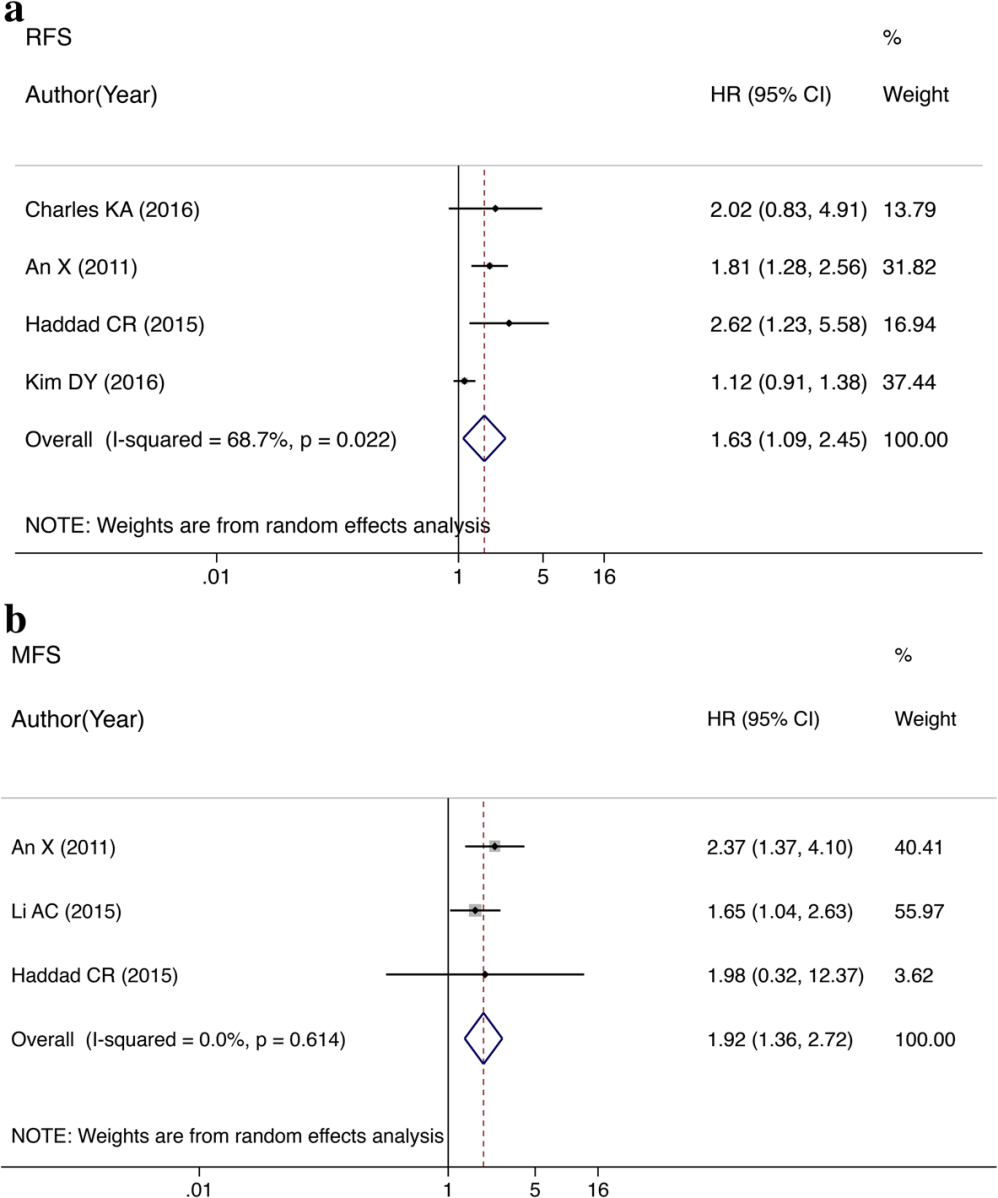
**a** Forest plots of studies evaluating HRs of the NLR on RFS for head and neck cancer. **b** Forest plots of studies evaluating HRs of NLR on MFS for head and neck cancer

The meta-analysis of RFS showed that the probability of tumor recurrence after treatment was greater in patients with high NLR than in those with low NLR. The difference was significant (HR = 1.63, 95% CI: 1.09–2.45; *P* = 0.017; heterogeneity, *I*^2^ = 68.7%, *P* = 0.022; Fig. 4a). There were three studies [27, 28, 39] that analyzed the correlation between the MFS and NLR. Patients with an elevated NLR had a higher probability of distant metastasis after treatment compared with those with a low NLR, the HR was 1.92 (95% CI: 1.36–2.72; *P* < 0.001; Fig. 4b). There was no statistically significant heterogeneity (*I*^*2*^ = 0.0%; *P* = 0.614).

### Publication bias and sensitivity analysis

**Fig. 5:**
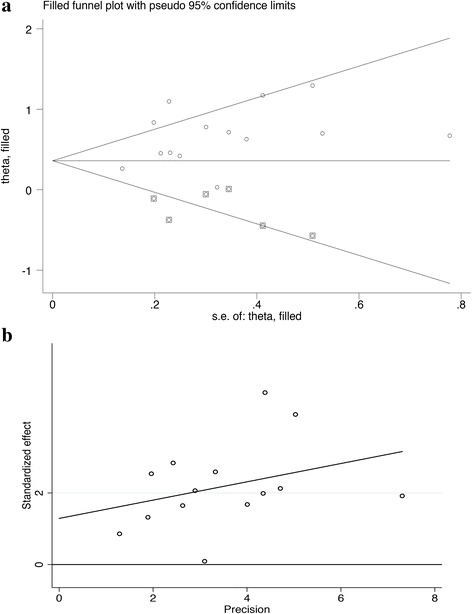
**a** Begg’s funnel plot with pseudo 95% confidence limits. **b** Egger’s publication bias plot

In the sensitivity analysis, the trim and fill method was used to combine six sets of data. The corrected data were consistent with the original results (HR = 1.459, 95% CI: 1.174–1.813; *P* = 0.001), indicating stable funnel plots of the meta-analysis (Fig. 5a). Publication bias was tested by the Egger test (*P* = 0.135), which indicated the absence of publication bias for OS (Fig. 5b).

## Discussion

This study was the first to evaluate the association of NLR in peripheral blood and prognosis in HNC patients. We found that patients with elevated pretreatment NLRs had predictable decreases in OS, DSS and PFS. Also, with increasing NLR cutoff value, mortality risk had a corresponding increasing trend, and patients were increasingly prone to local recurrence and distant metastasis. Our meta-analysis was consistent with previous studies of other malignant tumors. Other meta-analyses revealed better prognosis in patients with colorectal, non-small cell lung, stomach and prostate cancer who had low pretreatment NLR compared to those who had high NLR [15–19]. Pretreatment NLR reflects the status of systemic inflammation and the immune system. However, the cause of poor prognosis in HNC patients with elevated NLR requires further investigation.

Elevation of neutrophils reflects systemic as well as local inflammatory responses. Neutrophils provide a microenvironment conducive to the growth of tumor cells, and they promote tumor progression and invasion of malignant tumor cells [43]. Neutrophils produce and secrete tumor-promoting growth factors, such as epidermal growth factor, vascular endothelial growth factor, interleukin (IL)-6 and IL-8, that can promote tumor cell activation and facilitate tumor development, invasion and metastasis [8, 9]. In addition to producing cytokines, neutrophils secrete proteases, such as specific matrix metalloproteinases [44, 45], cysteine cathepsins [46, 47] and serine proteases [48]. These proteases can disrupt the connections between cells and degrade extracellular matrix and basement membrane proteins, thereby facilitating the migration of tumor cells [46–49]. They also promote epithelial cell proliferation, activate dormant tumor cells, and trigger revascularization [50], forming a link between inflammation and cancer. An increase in the number of neutrophils surrounding cancerous tissue can suppress antitumor immune responses while activating T lymphocytes and natural killer (NK) cells [51]. Thus, elevation of neutrophils and release of associated cytokines play a role in tumor metastasis and indicate poor prognosis in patients with malignant tumors.

In contrast, a reduction in the number of lymphocytes reflects decreased activity of lymphokine-activated killer cells [52], with inhibition of the monitoring of the host immune response [53]. The reduction of lymphocytes includes cells of the innate immune system, such as B lymphocytes, NK cells, CD4^+^ helper T lymphocytes and CD8^+^ cytotoxic T lymphocytes, leading to suppression of the immune response [7, 54]. Additionally, reduction of the number of lymphocytes results in decreased release of cytokines, such as interferon and tumor necrosis factor-α by tumor macrophages. These cytokines promote apoptosis of tumor cells, which is a key host defense against tumor cell invasion. The collective effect of these changes is attenuation of the antitumor-specific immune system [55, 56]. There is also a link between the immune system and systemic inflammation. Wong et al. [25] proposed that chronic inflammation is associated with increased myeloid-derived suppressor cells (MDSCs), which suppress the immune response. They also found that MDSC-mediated immune suppression resulted in dysfunction of the acquired (T cells) and innate (NK cells) immune systems; both of which play a major role in scavenging pathogens and mutant cells under normal conditions.

This study demonstrated that pretreatment NLR can be used to evaluate prognosis in HNC patients, but the optimum NLR cutoff value remains unclear. In the studies we analyzed, the NLR cutoff values ranged from 2.1 to 4.39 and were selected from the means of all patients in each study, or on the basis of previous research. Different studies used different cutoff values, making it difficult to perform the meta-analysis using a single, defined cutoff value. In order to obtain the optimal range of cutoff values, we divided the range into three equal groups for subgroup analysis using NLR cutoff values of 3.0 and 4.0, and a performed a meta-analysis of each subgroup. It is noteworthy that the increase in NLR resulted in similar mortality risks in subgroups 1 and 2, whereas the risk was significantly greater in subgroup 3 than in the other two subgroups. We infer that the prognostic value of NLR in HNC patients is influenced by a range of cutoff values. Optimally, we recommend using a continuous range of NLR values, rather than point values when selecting and comparing NLR cutoff values in future studies.

This meta-analysis had several limitations. First, all included studies were retrospective observational studies, and although multivariate analysis can control for confounding factors to a certain degree, selection bias was inevitable. Second, the NLR values could easily have been affected by infectious diseases, chronic infections, and use of glucocorticoid hormones that might have been present in the same period. Inflammation and NLR elevation are also believed to be associated with coronary heart diseases including acute coronary syndrome [57]. Interference of the NLR values by potential confounding factors associated with other diseases was thus inevitable. Third, NLR is closely associated with other variables associated with systemic inflammation, such as C-reactive protein and platelet-to-lymphocyte ratio. Interactions among these factors might have resulted in high collinearity in multivariate analysis by the Cox regression model, thereby influencing the evaluation of prognosis by NLR alone. Finally, there was a risk of reporting bias related to the method of retrieving full-text studies. Some studies did not report clinically significant results, and thus did not contribute to the calculated HR values, and some studies only included positive results in the data analysis.

## Conclusion

This meta-analysis showed that HNC patients with elevated pretreatment NLR had poor prognosis and were prone to local invasion and distant metastasis. NLR, which is easily obtained from peripheral blood samples, can help clinicians to determine the prognosis of HNC patients. Preoperative and postoperative interventions to regulate inflammatory and immune responses have a place in the long-term treatment of HNC, but future studies are required to validate the clinical use of NLR.

## Abbreviations

CIs: Confidence intervals
DFS: Disease-free survival
DSS: Disease-specific survival
HR: Hazard ratio
IL: Interleukin
MFS: Metastasis-free survival
Multi: Multivariate analysis
NLR: Neutrophil-to-lymphocyte ratio
NOS: Newcastle–Ottawa Scale
OS: Overall survival
PFS: Progression-free survival
RFS: Recurrence-free survival
